# Dementia and Traffic Accidents: A Danish Register-Based Cohort Study

**DOI:** 10.2196/resprot.6466

**Published:** 2016-09-27

**Authors:** Jindong Ding Petersen, Volkert Siersma, Connie Thurøe Nielsen, Mikkel Vass, Frans Boch Waldorff

**Affiliations:** ^1^ The Research Unit for General Practice Department of Public Health University of Southern Denmark Odense Denmark; ^2^ The Mental Health Services in the Region of Southern Denmark Department of Mental Health Kolding-Vejle Denmark; ^3^ The Research Unit for General Practice and Section of General Practice Department of Public Health University of Copenhagen Copenhagen Denmark; ^4^ The Research Unit for General Practice Department of Public Health University of Copenhagen Copenhagen Denmark

**Keywords:** dementia, accidents, traffic, comorbidity, epidemiology, public health

## Abstract

**Background:**

As a consequence of a rapid growth of an ageing population, more people with dementia are expected on the roads. Little is known about whether these people are at increased risk of road traffic-related accidents.

**Objective:**

Our study aims to investigate the risk of road traffic-related accidents for people aged 65 years or older with a diagnosis of dementia in Denmark.

**Methods:**

We will conduct a nationwide population-based cohort study consisting of Danish people aged 65 or older living in Denmark as of January 1, 2008. The cohort is followed for 7 years (2008-2014). Individual’s personal data are available in Danish registers and can be linked using a unique personal identification number. A person is identified with dementia if the person meets at least one of the following criteria: (1) a diagnosis of the disease in the Danish National Patient Register or in the Danish Psychiatric Central Research Register, and/or (2) at least one dementia diagnosis-related drug prescription registration in the Danish National Prescription Registry. Police-, hospital-, and emergency room-reported road traffic-related accidents occurred within the study follow-up are defined as the study outcome. Cox proportional hazard regression models are used for the main analysis.

**Results:**

Our study protocol has 3 phases including data collection, data analysis, and reporting. The first phase of register-based data collection of 853,228 individual’s personal information was completed in August, 2016. The next phase is data analysis, which is expected to be finished before December 2016, and thereafter writing publications based on the findings. The study started in January 2016 and will end in December 2018.

**Discussion:**

This study covers the entire elderly population of Denmark, and thereby will avoid selection bias due to nonparticipation and loss to follow-up. Furthermore, this ensures that the study results are reliable and generalizable. However, underreporting of traffic-related accidents may occur, which will limit estimation of absolute risks.

## Introduction

### Background

The elderly population is rapidly increasing globally as a result of extended life expectancy due to the success of social, medical, and economic development [1,2]. Consequently, ageing brings societal challenges due to the increase in the proportion of elderly people with chronic diseases including dementia [3].

Dementia is one of the major causes of functional disabilities and dependency among elderly people [4-6]. It is a symptomatic decline in cognitive ability severe enough to affect daily activities, and is usually irreversible, accumulative, and age-dependent [7]. Alzheimer disease and vascular dementia are the most common causes of dementia, other diseases and injuries to the brain are also contributing factors [8].

The prevalence of dementia is expected to increase substantially in parallel with elderly population growth [9]. By 2050, there will be a projected 135 million people suffering from dementia worldwide [3]. Denmark is facing the brunt of the dementia epidemic, an approximately 150,000 Danes are estimated to be afflicted with dementia by 2040, which is nearly doubled than of 2015 [10].

Elderly people in Denmark tend to choose cycling, walking, and private motor vehicles, as their daily mode of transportation [11]. Hence, leading society to a growing concern regarding road traffic-related accidents risk among those suffering from dementia in the coming years. The Statistics Denmark (DST) reported a total of 3375 road traffic-related accidents, including 182 fatal accidents occurring in 2014. Of those accidents, 15% of total and 32% of fatal accidents occurring among people aged 65 or older [12]. Dementia-related accidents within this group of people have never been well reported in Denmark.

However, older people do not necessarily pose an increased risk of traffic accidents as compared with other age groups, as age itself is a poor indicator of movement competence due to individual practice, experience, and general functioning skills being different [13]. However, dementia as a cognitive impairment disorder may affect vision, balance, judgement, perception, motor skills, and problem-solving, therefore it could increase the risk of traffic accidents [14]. Certain people with mild dementia however may still be capable of conducting themselves safely in traffic, at least for a certain period of time [15]. But, it is worth noting that motor skills are deteriorating, depending on the type and the severity levels of the dementia onset, and therefore the risk of accidents may vary [16,17].

Relatively limited numbers of studies have investigated dementia for the risk of traffic accidents, but with inconsistent results. Among published studies, approximately 2 to 10 times higher risks of crashes for people with dementia have been reported [18-21]. However, some others have reported no significant risk difference between people with dementia and their controls [22,23]. Despite traffic accidents being infrequent among elderly people, heterogeneity, small study population, and the quality of accident reporting, justify further studies into dementia and traffic accidents. Nationwide cohort studies with register-based information including exposures, confounders, and various health-related outcomes may fill these gaps.

A population-based Swedish study from 2013 with a sample size of more than 6.9 million people aged 20 years or older, found that the risk of total accidental deaths, including falls, suicides, transport accidents, and accidental poisoning, was 6- to 7-fold higher among people with dementia compared with the general population during 8 years of follow-up [24]. However, the Swedish study only measured accidental death, and therefore this study was not able to identify risk for nonfatal traffic accidents. Additionally, the Swedish study did not adjust for chronic illnesses and possibly harmful medications (eg, tranquillizers and sleeping medication) that may potentially affect the accidental risk [25].

Dementia is often comorbid with other chronic diseases, and the risk of having multiple morbidity increases with age [2,26]. In primary care in America, there is an average of 2.4 additional chronic diseases and above 5 prescribed medications associated with dementia patients aged 65 or older [27]. In Scotland, only 5.5% people with dementia had no other chronic diseases [28]. Sharing some common risk factors and pathophysiological mechanisms such as inflammation and endothelial dysfunction may be one of the reasons for coexistence of multiple chronic diseases, other factors may also play a role [29].

National prevalence of dementia-related comorbidity in the elderly population in Denmark is unclear. However, depression, Type 2 diabetes (T2D), ischemic heart disease (IHD), and chronic obstructive pulmonary disease (COPD), are among the 10 most common chronic diseases among Danish patients, and have been found as independent risk factors for the development of dementia, and can even exacerbate dementia [30-37]. For instance, a systematic review with 17 cohort studies reported that depression, especially late-life depression, was associated with a significant risk of dementia (pooled risk=1.59, 95% confidence interval (CI)=1.41-1.80) [32]. Another meta-analysis with 19 population-based longitudinal studies found a 2- to 3-fold higher risk of developing dementia with diabetes [38].

Moreover, these chronic diseases have also been reported as independent risk factors for traffic accidents [24,39-41]. A structure review with 7 studies found increased odds, or risk ratios of crashes ranging from 1.9 to 7.7 for people with post stroke [42]. Given that the combined effects of those chronic diseases are higher than single or additive effects [43,44], it is possible that dementia accompanied with other chronic disorders can pose an even higher risk of traffic accidents, and chronic disease-related medications may modify the risk estimations. However, to date, those issues have not been studied in much detail elsewhere, or in Denmark.

### Aims of the Study

This study protocol overall aim is to investigate the risk of road traffic-related accidents for people aged 65 years or older with diagnosis of dementia in Denmark. The following will be investigated: (1) the risk of road traffic-related accidents among older people with and without dementia, (2) the effect modification of dementia and association with the risk of road traffic accidents by comorbidities, and (3) the effect modification of dementia and association with the risk of road traffic accidents by sedative prescription medications.

## Methods

### Study Design and Population

The study is designed as a register- and population-based cohort study consisting of all residents in Denmark aged 65 years or older as of January 1, 2008 (n=853,228). These people are followed for 7 years from baseline until December 31, 2014 to assess the incidence of road traffic-related accidents attributable to a dementia diagnosis. The follow-up period of 7 years is chosen because (1) the median survival time for people with dementia in Denmark is 6.6 years; this is slightly longer than other countries (median ranged 3.2-6.6 years) [45,46], and (2) the validity of the dementia diagnosis in recent years has increased and therefore is more accurate from 2008 compared with earlier registration [47-50].

### Data Sources

Personal-level data are available in the Danish Civil Registration System (CRS) [51]. This registry electronically records the name, address, migration, marital status, date of birth, place of birth, date of death, and other basic information on all residents in Denmark since 1968. Using a unique 10-digit Civil Personal Register (CPR) number assigned to each individual at birth, or to a person who holds a Danish residence upon immigration, one can access an individual’s information in all national registers, hospitals, general practitioners, police offices, and other authorities in Denmark.

### Assessment of Dementia and Comorbidity

Dementia and subtype of dementia including Alzheimer’s disease [52], vascular dementia [53], frontotemporal dementia [54], dementia with Lewy bodies [55], mixed dementia [56], Parkinson’s disease [57], and dementia without specification, are identified by any primary and secondary diagnosis in the Danish National Patient Register (NPR) (Table 1), or in the Danish Psychiatric Central Research Register (PCRR) [58,59].

International Classification of Diseases versions 10 codes (ICD-10) are used for disease classification in the registers. Additionally, the prescription of antidementia drugs and the corresponding Anatomical Therapeutic Chemical (ATC) codes (Multimedia Appendix 1) from the Danish National Prescription Registry (DNPR) are also used to identify people with dementia disorders [60].

To more accurately define the earliest date of a dementia diagnosis, the diagnosis is dated back to either the first inpatient or outpatient record ever mentioning dementia or the first prescription of antidementia medication since the inception of the DNPR, whichever comes first. A person is identified with dementia if this person meets at least one of the following criteria: (1) a diagnosis of disease in NPR or in PCRR, and/or (2) at least one antidementia drug registration in DNPR.

**Table 1:** *International Classification of Diseases*, Tenth Revision (ICD-10) codes for dementia and other chronic disease^a^diagnoses in Danish health registers.

Diseases		ICD-10 codes
**Dementia**		
	Alzheimer’s disease	F00.0, F00.1, F00.2, F00.9, G30.0, G30.1, G30.8, G30.9
	Vascular dementia	F01.0, F01.1, F01.2, F01.3, F01.8, F01.9
	Frontotemporal dementia	F02.0
	Dementia with Lewy bodies	G31.83
	Mixed dementia	
	Parkinson’s disease	F02.08
	Dementia without specification	F03.9
**Other chronic diseases**		
	Type 2 diabetes	E11
	Chronic obstructive pulmonary disease	J44
	Ischemic heart disease	I20–I25
	Depression	F32-F33
	Hypertension	I10, I15
	Stroke	I60-I69
	Atrial fibrillation	I48
	Asthma	J45

^a^The inclusion criteria for the chronic illness are based on the 10 most common chronic conditions among Danish patients [61], as well as prior studies that found that those diseases are both linked to dementia [62-67], and traffic accidents [42,68].

In this study, comorbidity is defined as a person with at least 2 chronic diseases as listed in Table 1. For chronic disease ascertainment, we apply a similar assessment procedure as with dementia. A person is identified with a specific chronic disorder if this person had a diagnosis of disease in NPR or in disease-specified register if available (Multimedia Appendix 2). Because there is no such registration regarding the exact time when the chronic disease symptoms began, we use the date of registration and/or drug prescription as the initial time point for dementia onset and the comorbidity. Therefore, if the date of registration for any of the comorbidities has been recorded prior to the date of dementia registration, we consider the comorbidity has occurred before onset of dementia, and vice versa.

### Study Outcomes Ascertainment

The primary outcome (time to first road traffic-related accident event) is Danish police-reported road traffic accidents including minor injuries, serious injuries, and fatal accidents caused by road traffic during the study follow-up interval. The information such as the date, location, and type of traffic accident is recorded in DST. Because some traffic injuries may only be registered by the hospital or emergency room without being reported to the police, any hospital or emergency room diagnosis representing road traffic accident (ICD-10 codes V00-V89, V98-V99) within the study follow-up period in NPR is supplementary being assessed.

### Other Covariates

Age, sex, education, marital status, geographic location, and living in a nursing home are examined as predictors and adjustment variables based on prior knowledge in the present study. Those data are available in CRS and in DST. Driving experience (eg, the years of holding a valid driver license) as one of the potential confounders will also be included in the sensitivity analysis if possible. The chronic disease-related medications that impair ability to drive are addressed as covariates in our study and ascertained by the information of side effects from the drug labelling (Multimedia Appendix 3). If medication involvement in the analysis gets too complicated, we will begin with the most common drugs for the listed chronic diseases.

### Statistical Analyses

For aims 1, 2, and 3, the relation between a diagnosis of dementia, or of selected comorbid chronic diseases, as well as antidementia medication, and the incidence of traffic accidents, will be analysed in Cox proportional hazard (Cox) regression models. The total period at risk for a person is the time from January 1st, 2008 until the first occurrence of a traffic accident, death, emigration or end-of-follow-up at December 31st, 2014, whichever comes first; the latter 3 occurrences are censoring events. Dementia, and the other selected comorbid conditions, will be modelled as time-varying covariates in that persons contribute to “nondiagnosed” person-years before and “diagnosed” person-years after the first occurrence of the corresponding diagnosis.

The magnitude of the associations will be reported as hazard ratios (HRs) with 95% CIs. The associations of dementia and other selected comorbid conditions with a traffic accident incidence will be adjusted for several potential confounders in multivariable Cox regression models: age, sex, education, marital status, geographic location, living in a nursing home, medication, and Charlson comorbidity index (CCI) [69]. The proportional hazard assumption will be evaluated by adding interaction terms between the logarithm of time and the independent variables to the model; a joint test for these interaction terms evaluates the proportional hazard assumption.

By the addition to the model of interactions between the selected chronic disorders and the dementia diagnosis, we will investigate a possible differential effect of dementia on the incidence of traffic accidents depending on the aforementioned factors. The different impacts of dementia for the different levels of the corresponding interacting variable will be recorded, and the differences will be evaluated for statistical significance.

### Study Power Calculation

There were 6323 road traffic accidents (including injuries and fatalities) in 2008 in Denmark, which represented 1.16‰ (6323/5,475,791) of the total population (DST 2008). If we assume that there is a 1% prevalence of dementia in the Danish population (which is lower than the 1.53% that Alzheimer Europe has estimated for 2012) [70], we can, with nationwide data, detect a traffic accident incidence increase from 1.16‰ for those without dementia to 1.60‰ for those with dementia with 80% power and 5% significant level.

There were 661 road traffic accidents in 2008 among people aged 65 years and over in Denmark, which represented 0.78‰ (661/853,041) of people aged 65 and over in Denmark (DST 2008). If we assume that there is a 7% prevalence of dementia in this age group [10,71,72], we can, with these data, detect a traffic accident incidence increase from 0.78‰ for those without dementia to 1.13‰ for those with dementia with 80% power and 5% significant level.

All statistical tests will be two-sided and use a significance alpha level of 5%. STATA 14.0 will be used for all statistical analysis.

### Ethical Consideration and Dissemination

The study protocol was approved by the Danish Data Protection Agency for data permission as well as for ethical considerations (J.no. 2016-41-4674).

## Results

**Figure 1:**
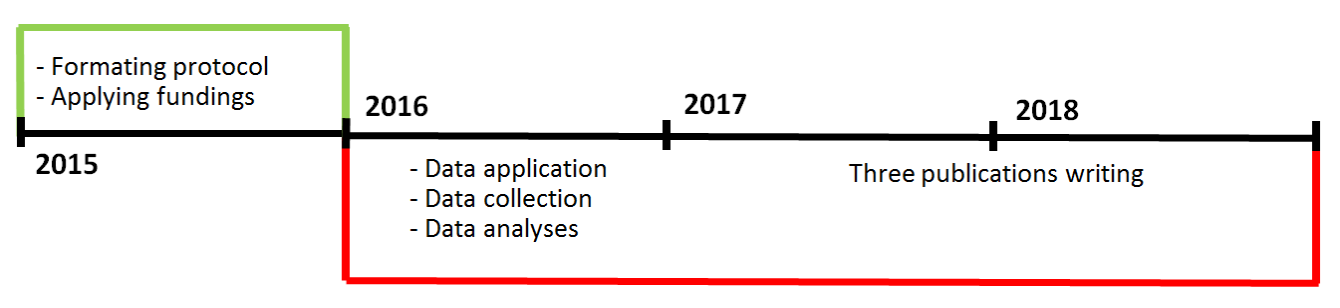
The study timeline.

This 3-year PhD study is planned from January 1, 2016 to December 31, 2018. The study timeline is illustrated in the Figure 1. At the current stage, we have finished the data collection, and are beginning the data analysis.

Three publications are planned within the protocol. The publication titles at the current stage can potentially be: (1) The risk of road traffic-related accidents among people with dementia, (2) The modification of the dementia and association with the risk of road traffic accidents by comorbidities, or (3) The modification of the dementia and association with the risk of road traffic accidents by sedative prescription medications.

## Discussion

### Strengths and Limitations

This is the first study to evaluate elderly people with dementia and the link to transport safety injuries in Denmark. With our objectives, we are expecting to find an association between the onset of dementia and the risk of traffic-related accidents among people aged 65 or older.

Denmark is internationally recognized for having rigorous registrations of data regarding many activities. Using nationwide register data to examine the association between dementia and the risk of traffic-related accidents with an entire national elderly population is a major strength of our study, as selection bias due to nonparticipation or loss to follow-up is negligible [47]. The health care system is free of charge and all citizens have equal access to it. Hence, our study results are more reliable and generalizable than those of previous studies with limited sample sizes or case-control designs.

However, underreported traffic-related accidents might occur, and this may limit our risk estimation. It seems a common social norm is that, with very minor traffic accidents such as scratches, the drivers tend to negotiate between each other rather than to rush to report to the police. Nevertheless, using police and hospital, as well as emergency room-registered road traffic accidents, the present study has much more complete data than previous studies on a similar topic.

### Implications

Transportation in an ageing society is a general challenge, and it is considered appropriate to actively engage people with dementia and their families in social chores. But the traffic risk has not been well assessed. Therefore, this study may identify the magnitude of traffic accident risk among people with dementia in order to provide initiatives for reducing this potential risk.

## Appendix

Multimedia Appendix 1 ATC-codes for drugs in the Danish National Prescription Registry.

Multimedia Appendix 2 Overview of databases and indicators for data assessment.

Multimedia Appendix 3 Medicines labelled with side-effects as impaired driving ability.

## Abbreviations

ATC: anatomical therapeutic chemical
CCI: Charlson comorbidity index
CI: confidence interval
COPD: chronic obstructive pulmonary disease
Cox: Cox proportional hazard
CPR: Civil Personal Register
CRS: Danish Civil Registration System
DNPR: Danish National Prescription Registry
DST: Statistics Denmark
HRs: hazard ratios
ICD-10: *International Classification of Diseases, Tenth Revision*
IHD: ischemic heart disease
NPR: Danish National Patient Register
PCRR: Danish Psychiatric Central Research Register
T2D: type 2 diabetes
