# Supplementary material for: Dementia and Traffic Accidents: A Danish Register-Based Cohort Study
Source: JMIR Res Protoc. 2016 Sep 27;5(3):e191. doi: 10.2196/resprot.6466 (PMC5059484; doi:10.2196/resprot.6466)
Supplement: Multimedia Appendix 1 [file resprot_v5i3e191_app1.pdf]

Appendix A: ATC-codes for drugs in the Danish National Prescription Registry

Diseases	Prescription name	Drug	ATC-codes
Dementia	Anticholinesterases	Tacrine, Donepezile, Rivastigmine, Galantamine	N06DA01, N06DA02, N06DA03, N06DA04
	Other anti-dementia drugs	Memantine	N06DX1
Type 2 diabetes	Anti-diabetic		A10B
Depression	SSRI (selective serotonin re-uptake inhibitors)	Fluoxetine, citalopram, paroxetine, sertraline, fluvoxamine, and escitalopram	N06AB
	MAOIs (monoamine oxidase inhibitors)	Isocarboxazid, moclobemide	N06AF, N06AG
	Other antidepressants	Mianserin, nefazodone, mirtazapine, venlafaxine, reboxetine, duloxetine, and agomelatine	N06AX
	Tricyclic antidepressants (TCAs)	Desipramine, imipramine, imipramine oxide, clomipramine, opipramol, trimipramine, Iofepramine, dibenzepin, amitriptyline, nortriptyline, protriptyline, doxepin, iprindole, melitracen, butriptyline, dosulepin, amoxapine, dimetacrine, amineptine, maprotiline, quinupramine	N06AA
COPD		Inhaled corticosteroids with long-acting beta2-agonists (ICS/LABA);<br>Long-acting anti-cholinergics (LAMA);<br>Long acting beta$_2$-agonists (LABA)(51)	R03A (adreergics for inhalation) R03B (other drugs for obstructive lung disease, inhalation)
Ischemic heart disease	Anti-IHD medicine(52)	Cardiac glycosides, Vasodilators, Antihypertensiva, Diuretics, Alpha-and beta-blockers, Calcium channel blockers, ACE-inhibitors and angiotensin-II antagonists, Lipid-modifying agents	C01A, C01D, C02, C03, C07, C08, C09, C10
Hypertension	Anti-hypertensive medicines(53)	α-antagonists, Diuretics, Beta-blockers, Calcium antagonists, ACE-inhibitors, and angiotensin II receptor antagonists	C02, C03, C07A, C08, C09
Stroke	Antiplatelet(54, 55)	Acetylsalicylic acid, Dipyridamole, Low-dose aspirin, dipyridamole, Clopidogrel	B01AC06, N02BA01, B01AC07, B01AC30, B01AC04
Atrial fibrillation	Anti-arrhythmic medication(56)	Vitamin K antagonists, Aspirin; β-blockers; Non-dihydropyridine calcium-channel blockers, Amiodarone, and Digoxin	B01AA03, B01AA04, B01AC06, C08
Asthma	Anti-asthmatic medication(57)	SABAs; Anticholinergics Glucocorticosteroids; LABAs; Fixed	R03AC02, R03AC03, R03AC04, R03AC12, R03CC03, R03CC02, R03BB01, R03BA01, R03BA02, R03BA05, R03BA07, R03BA12, R03BA13, R03AC12, R03AC13, R03DC03, R03DC04, R03CC12, R03BB04, R03AK03, R03AK04, R03AK06, R03AK07
