# Supplementary material for: Dementia and Traffic Accidents: A Danish Register-Based Cohort Study
Source: JMIR Res Protoc. 2016 Sep 27;5(3):e191. doi: 10.2196/resprot.6466 (PMC5059484; doi:10.2196/resprot.6466)
Supplement: Multimedia Appendix 2 [file resprot_v5i3e191_app2.pdf]

Appendix B. Overview of databases and indicators for data assessment

Type of data	Available databases	Indicators
Personal information	The Danish Civil Registration System	Age, sex, marital status, vital status, geographic location, emigration.
Dementia	The Danish National Patient Register;<br>The Danish Psychiatric Central Research Register;<br>The Danish National Prescription Registry.	Date of diagnosis,<br>Prescriptions of anti-dementia drugs.
Type 2 diabetes	The Danish National Patient Register;<br>The Danish National Diabetes Register;<br>The Danish National Prescription Registry.	Date of diagnosis,<br>Prescriptions of anti-diabetic drugs
Ischemic heart disease	The Danish National Patient Register;<br>The Danish National Heart Register;<br>The Danish National Prescription Registry.	Date of diagnosis,<br>Prescription of heart-related drugs
COPD	The Danish National Patient Register;<br>The Danish National Prescription Registry.	Date of diagnosis,<br>Prescription of anti-COPD drugs
Depression	The Danish National Patient Register;<br>The Danish National Prescription Registry.	Date of diagnosis,<br>Prescription of anti-depressive drugs
Medications	Danish National Prescription Registry	Date of prescriptions of medicine
Traffic accident	Statistics of Denmark;<br>The Danish National Patient Register;	Date of accident, location, type of accident.<br>Date of diagnosis and hospitalized
Driving license	Denmark Police Office	Date of issued, date of license cession.
Other variables	Statistics of Denmark	Education level; status of living in a nursing home
