# Supplementary material for: Dementia and Traffic Accidents: A Danish Register-Based Cohort Study
Source: JMIR Res Protoc. 2016 Sep 27;5(3):e191. doi: 10.2196/resprot.6466 (PMC5059484; doi:10.2196/resprot.6466)
Supplement: Multimedia Appendix 3 [file resprot_v5i3e191_app3.pdf]

Appendix C: Medicines labelled with side-effects as impaired driving ability

Diseases				
**Digestive system** (Fordøjelsessys temet)	Peristaltikfremmende (A03FA)	Metoclopramid	A03FA01	Primperan, Emperal, Gatro-Timelets, Akineton, Lysantin, Kemadrin
	Antikolinergikum med kvartært nitrogenatom (A03)	Hyoscinbutylbromid,<br>Propanthelinbromid	A03BB01,<br>A03AB05	Buscopan<br>Ercoril
**Ulcer Medicine** (Mavesårsmedi cin)	H2-receptor-antagonister (A02BA)	Ranitidin	A02BA02	Acikure
**Heart** (Hjerte-Kar)	Glykosider (C01AA)	Digoxin	C01AA05	Digoxin
	Beta-blokker (C07A)			
	Non-selektive (C077A)	Propranolol<br>Pinidol<br>Sotalol	C07AA05<br>C07AA03<br>C07AA07	Propol, Propranolol, Propranolol Retard.<br>Hexapindol, Visken.<br>Sotalol.
	Beta1-selektive (C07AB)	Atenolol	C07AB03	Atenodan, Atenolol, Tenormin, Uniloc.
		Bisoprolofumarat		Bisoprolol, Cardicor, Emconcor.
		Esmolol	C07AB09	Brevibloc
		Meteprolol	C07AB02	Mepronet, Metocar, Metomylan, Metoprolol.
		Nebivolol	C07AB12	Hypoloc, Lobivon, Nebivolol.
	Beta-blokkere med alfa-blokerende effekt (C07B)	Atenolol, Chlortalidon	C07CB03	Tenidon, Tenoretic.
		Hydrochlorthiazid, Nebivolol	C07BB12	Hypoloc
		Hydrochlorthiazid, Metoprolol	C07BB	Zok-Zid
**Drugs for incontinence** (Midler mod inkontinens)	Antikolinergika (G04BD)	Tolterodin	G04BD07	Detrusitol Retard, Tolterodin, Tolterodine, Tolterodintartrat.
		Solifenacin	G04BD08	Vesicare
		Solifenacin, Tamsulosin	G04CA53	Urizia, komb.
		Fesoterodin	G04BD11	Toviaz
**Analgesics** (Analgetika)	Opioider, kombinationer (N02AA)	Dimethylaminodiphenylbuten,<br>Ketobemidon	N02AG02	Ketogan
	Rene opiodagonister	Oxycodon<br>Morphin	N02AA05<br>N02AA01	Oxycontin<br>Morfin
	NSAID (M01A)			
	ASA (N02BA)			
	COX-hæmmere (B01AC)			
**Sleep/Anxiety** (Søvn/Angst)	Quinin (P01BC01)			
**Hypnotica** (Hypnotica)	Sederende antihistaminer (R06A)	Promethazin	R06AD02	Phenergan, Prometazin
	Benzodiazepiner (hypnotica) (N05CD)	Lormetazepam<br>Midazolam<br>Nitrazepam<br>Triazolam	N05CD06<br>N05CD08<br>N05CD02<br>N05CD05	Pronoctan<br>Midazolam<br>Nitrazepam, Pacisyn<br>Halcion
	Benzodiazepinlignende midler, Cyclopyrroloner (N05CF)	Zopiclon	N05CF01	Imovane, Sonata, Zonoct<br>Imozop, Zopiclone
**Anxiolytics** (Anxiolytica)	Imidazopyridiner	Zolpidem	N05CF02	Stilnoct, Zolpidem, Zonoct, Imoclone
	Pyrazolopyrimidiner	Zaleplon	N05CF03	Sonata
	Benzodiazepiner (Anxiolytica) (N05BA)	Alprazolam<br>Bromazepam<br>Diazepam<br>Chlordiazepoxid<br>Clobazam<br>Lorazepam<br>Oxazepam	N05BA01<br>N05BA08<br><br>N05A02<br>N05BA09<br>N05BA06<br>N05BA04	Alprazolam, Alprox, Tafil<br>Bromam, Lexotan<br>Stesolid,Apozepam,Hexalid, Diazepam<br>Kopoxid, Risolid<br>Frisium<br>Lorazepam, Orfidal, Temesta<br>Alopam, Oxabenz, Oxapax, Oxazepam
**Psychiatric drugs** (Psykofarmaka)	Buspiron (Azapirongruppen) (N05BE)	Busporion	N05BE01	Busporion, Busporione
	Tricykliske antidepressive (N06AA)	Amitriptylin<br>Clomipramin<br>Dosulepin<br>Doxipen<br>Imipramin<br>Maprotilin<br>Nortriptylin	N06AA09<br>N06AA04<br>N06AA16<br>N06AA12<br>N06AA02<br>N06AA21<br>N06AA10	Amytriptylin, Saroten<br>Anafranil, Clomipraminhydrochlorid, Klomipramin<br>Prothiaden<br>Sinquan<br>Imipramin<br>Ludiomil<br>Noritren

	Antipsykotika<br>(N05A)	**1.generations. Lavdosis**<br>Flupentixol<br>Haloperidol<br>Pimozid	 N05AF01<br>N05AD01<br>N05AG02	 Fluxanol<br>Serenase<br>Orap
		**1.generations. Middeldosis**<br>Periciazin<br>Prochlorperazin<br>Zuclopenthixol<br>Zuclopenthixoacetat	 N05AC01<br>N05AB04<br>N05AF05<br>N05AF05	 Neulactil<br>Stemtil<br>Cisordinol, Clopixol<br>Cisordinalacutard, Zuclopenthixoacetat
		**1.generations. Højdosis**<br>Chlorprothixen<br>Levomepromazin<br>Melperon<br>Pipamperon<br>Sulpirid	 N05AF03<br>N05AA02<br>N05AD03<br>N05AD05<br>N05AL01	 Truxal<br>Nozinan<br>Buronil<br>Dipiperon<br>Dogmatil
		**2.generations**<br>Amisulprid<br>Aripipazol<br>Asenapin<br>Clozapin<br>Olanzapin<br>Paliperidon<br>Quetiapin<br>Risperdon<br>Sertindol<br>Ziprasidon	 N05AL05<br>N05AX12<br>N05AH05<br>N05AH02<br>N05AH02<br>N05AX13<br>N05AH04<br>N05AX08<br>N05AE03<br>N05AE04	 Amisulprid, Solian.<br>Abilify<br>Sycrest<br>Clozapin, Clozapine, Leponex.<br>Olanzapin, Olanzapine, Zalasta, Zyprexa<br>Invega<br>Alzen, Quetiapin, Quetiapine, Seroquel, Stadaquel.<br>Risperanne, Risperdal, Risperidon.<br>Serdolect<br>Geodon,Zeldox,Ziprasidon,Ziprazidone
**Anti-Parkinson agents**<br>(Antiparkinson smidler)	Dopaminagonister<br>(N04)	**Ergot dopaminagonister**<br>Bromocriptin<br>Cabergolin	**N04BC**<br>N04BC01<br>N04BC06	 Parlodel<br>Cabaser
		**Non-ergot dopaminagonister**<br>Apomorfin<br>Pramipexol<br>Ropinirol<br>Rotigotin	 N04BC07<br>N04BC05<br>N04BC04<br>N04BC09	 APO-go, Apomorphin<br>Derinik,Mirapexin,Oprymea,Pramipexol,Pramipexole,Sifrol<br>Adatrel,ReQuip,Ropinirol<br>Neupro
**Epilepsy Medications**<br>(Epilepsimedicin)	Antikolinergika<br>(N04A)	Biperiden<br>Orphenadrin<br>Procyclidin	N04AA02<br>N04AB02<br>N04AA04	Akinetom<br>Lysantin<br>Kemadrin
	Barbitursyrederivater<br>(N03AA)	Phenobarbital<br>Primidon	N03AA02<br>N03AA03	Fenemal<br>Primidon
	Benzodiazepiner<br>(epilepsi) (N03AE, N05BA)	Clobazam<br>Clonazepam<br>Diazepam<br>Midazolam	N05AB09<br>N03AE01<br>N05BA01<br>N05CD08	Frisium<br>Rivotril<br>Stesolid<br>Buccolam
	Oxicarbazepin<br>(N03AF)	Carbamazepin<br>Oxcarbazepin<br>Eslicarbazepin<br>Rufinamid	N03AF01<br>N03AF02<br>N03AF04<br>N03AF03	Karbamazepin,Tegratal Retard,Tegretol,Trimonil<br>Apydan,Oxacarbazepin,Trileptal<br>Zebinix<br>Inovelon
	Gabapentin (N03AX)	Gabapentin<br>Lacosamid<br>Lamotrigin<br>Levetiracetam<br>Perampanel<br>Pregabalin<br>Stiripentol<br>Retigabin<br>Topiramat<br>Zonisamid	N03AX12<br>N03AX18<br>N03AX09<br>N03AX14<br>N03AX22<br>N03AX16<br>N03AX17<br>N03AX21<br>N03AX11<br>N03AX15	Gabapentin, Neurontin.<br>Vimpat<br>Lamictal, Lamotrigin.<br>Levetiracetam, Keppra<br>Fycompa<br>Lyrica<br>Diacomit<br>Trobalt<br>Topimax, Topiramat<br>Zonegran
	Phenytoin og fosphenytoin<br>(N03AD)	Ethosuximid	N03AD01	Zarondan
	N03AG	Valproat<br>Vigabatrin	N03AG01<br>N03AG04	Delepsine, Orfiril.<br>Sabrilex
	Carbonanhydraasehæmmere (S01EC)	Acetazolamid		
